# Atrial Fibrillation Validation Among Patients with Ischemic Stroke: An Electrocardiogram Comparison Between 24 h Holter and Single-Lead Wearable Devices: Rationale and Design of the AVANT-GARDE Trial

**DOI:** 10.3390/biomedicines13112677

**Published:** 2025-10-31

**Authors:** Yerim Kim, Jong-Ho Park, Yong-Jae Kim, on behalf of the AVANT-GARDE Investigators

**Affiliations:** 1 Department of Neurology, Kangdong Sacred Heart Hospital, Hallym University College of Medicine, Seoul 05355, Republic of Korea; brainyrk@hallym.ac.kr; 2 Department of Neurology, Eunpyeong St. Mary’s Hospital, The Catholic University of Korea, Seoul 03312, Republic of Korea; neurotector.jhp@gmail.com; 3 Kim’s Neurology Clinic, Seoul 05854, Republic of Korea

**Keywords:** atrial fibrillation, embolic stroke of undetermined sources, ischemic stroke

## Abstract

**Introduction/Aim:** As the prevalence of atrial fibrillation (AF) increases with the aging population, the challenge of enhancing the detection rate of AF in embolic stroke of undetermined source (ESUS) has intensified. AF is often detected only after a debilitating or fatal cardioembolic stroke, underscoring the crucial need for early identification to prevent further ischemic stroke (IS). This study aims to assess the efficacy of AF detection in patients with ESUS using a single-lead patch (mobiCARE™; Seers Tech, Inc.) over a period of at least 72 h and up to 5 days, in comparison with standard 24 h Holter monitoring. **Design:** This multicenter, prospective, consecutive observational trial involves patients aged 18 years and older who have experienced an IS within the past six months. **Study outcomes**: The primary outcome is the initial detection of AF using either a single-lead wearable patch or 24 h Holter monitoring at both baseline and at a 6-month follow-up among patients diagnosed with ESUS stroke. Secondary outcomes include the first detection of AF among stroke patients with large-artery atherosclerosis or small-vessel occlusion, particularly those whose transthoracic echocardiography reveals a left atrial size of ≥44 mm, an echocardiographic marker of AF. **Discussion:** Prolonged cardiac monitoring increases the detection rate of paroxysmal AF, but the optimal method should be non-invasive, patient-friendly, and accurate. This new technology may reduce patient burden and socioeconomic impact by facilitating AF detection and preventing cardioembolic stroke, even in patients with non-cardiogenic stroke.

## 1. Introduction

The growing prevalence of atrial fibrillation (AF) coincides with the global increase in the elderly population and ranges from 0.4% to 2% among adults [1]. AF, a common arrhythmia, often becomes apparent only after a severe or fatal cardioembolic event. Conventional Holter monitoring, particularly in cases without a clearly identified high-risk cardioembolic source, demonstrates low AF detection rates. Moreover, in patients with traditional atherogenic stroke, recurrent stroke is often associated with AF-related pathologies [2]. Therefore, the early detection of AF is critical for the effective prevention of recurrent events.

In approximately one-third of ischemic stroke (IS) cases, there is no immediately identifiable embolic cause, leading to classification as an “embolic stroke of undetermined source” (ESUS). Notably, over 30% of ESUS patients are found to have underlying paroxysmal atrial fibrillation (pAF) [3]. However, the rate of AF detection via short-term ECG monitoring in these patients is only about 2–3%, suggesting that AF frequently remains undetected. Long-term monitoring using implantable loop recorders (ILRs) achieves detection rates of approximately 23–30%. Although the 2023 European Stroke Organization (ESO) guidelines recommend more than 48 h of continuous cardiac monitoring with an ILR for AF screening in all patients with IS and ESUS [4], its invasive nature limits widespread use [5]. Consequently, echocardiographic indicators such as left atrial (LA) enlargement and laboratory markers like NT-proBNP are used as supplementary tools for AF detection.

The 2020 guidelines from the European Society of Cardiology recommend the use of wearable devices for single-lead ECG recording, with a Class Ia recommendation for AF diagnosis confirmation [6]. Furthermore, a comprehensive systematic review and meta-analysis, which included 47 studies, supports a non-invasive rhythm monitoring strategy as an initial approach before adopting invasive techniques [7]. Based on these findings, a non-invasive single-lead wearable monitoring device may be more sensitive and effective for AF diagnosis than traditional Holter monitoring, thereby reducing the need for repeated ECG tests.

This study aims to evaluate the efficacy of AF detection using a single-lead patch compared to conventional 24 h Holter monitoring in stroke patients identified with ESUS. Additionally, we aim to explore the detection rates of incidental AF in patients exhibiting LA enlargement or elevated NT-proBNP levels, providing potential indicators of AF in patients with non-cardioembolic stroke, including those due to large-artery atherosclerosis (LAA) and small-vessel occlusion (SVO).

## 2. Design

### 2.1. Trial Design

The AVANT-GARDE trial, a multicenter, prospective, observational trial (registration number cris.nih.go.kr KCT0008110), is designed to detect AF in patients with IS using the single-lead wearable patch and comparing it with 24 h Holter monitoring.

### 2.2. Rationale for Investigation

Earlier studies investigating the effectiveness of non–vitamin K-dependent oral anticoagulants (NOACs) and aspirin in ESUS patients revealed ambiguities regarding eligibility criteria, potentially including patients with atherothrombotic origins, thereby leading to inconclusive outcomes [8,9,10]. To improve the precision and specificity of the inclusion criteria related to cardioembolic mechanisms, we incorporated additional diagnostic evaluations such as brain imaging markers (embolic imaging patterns), transthoracic echocardiographic (TTE) indicators (LA size and E/e′ ratio), and serum markers (NT-proBNP) [11].

### 2.3. Definition of ESUS

ESUS is defined as an embolic stroke that is non-lacunar (with a lesion size greater than 1.5 cm on CT or greater than 2 cm on magnetic resonance imaging [MRI]) and non-atherosclerotic, without proximal arterial stenosis (>50%) in the culprit artery or known high-risk cardioembolic sources [12]. Embolic imaging patterns include multiple infarcts, simultaneous involvement of different circulations, and isolated cortical infarcts [13].

### 2.4. Study Population

Nine hospitals in South Korea participated in this trial: Eunpyeong St. Mary’s Hospital, the Catholic University of Korea, Kangdong Sacred Heart Hospital, Gachon University Gil Medical Center, Hanyang University Myongji Hospital, SMG–SNU Boramae Medical Center, Soon Chun Hyang University Hospital Bucheon, Inje University Sanggye Paik Hospital, and Chosun University Hospital. Each institution is equipped with a dedicated stroke unit and the necessary monitoring and imaging facilities to provide standardized stroke care by experienced specialists.

The absence of diagnosed AF at admission, confirmed through baseline surface ECG, is the key criterion. Eligible participants are as follows: (1) age over 18 years, (2) non-lacunar infarction or non-atherosclerotic cerebral infarction without corresponding artery stenosis > 50% (less than 6 months after stroke onset), (3) no history of AF and showing no AF during initial monitoring, (4) at least 18 h of recording on 24 h Holter monitoring, (5) at least 72 h of recording on a single-lead wearable patch, and (6) who underwent MRI and TTE examination. Subjects were excluded if they (1) exhibited AF on baseline ECG, (2) had valvular heart diseases (e.g., rheumatic mitral stenosis, a mechanical or bioprosthetic heart valve, or mitral valve repair, infective endocarditis), (3) had underlying disease related to AF (recent cardiothoracic and noncardiac surgery (<2 weeks), recent ST-elevation myocardial infarction (MI) (<3 months), pericarditis, myocarditis, hyperthyroidism, electrocution, pneumonia, and pulmonary embolism), (4) had implanted pacemakers, (5) suffered from advanced systemic disease or coexisting neurological/psychiatric conditions, (6) had contact dermatitis or other skin disorders, (7) lacked data on transesophageal echocardiography, or (8) were unable to participate in the study due to other causes.

According to the TOAST classification system, large artery atherosclerosis is defined as corresponding artery stenosis > 50% and small-vessel disease as a subcortical lesion < 15 mm. Patients whose index stroke was LAA or SAO were included if they exhibited LA enlargement.

### 2.5. Transthoracic Echocardiography

Transthoracic echocardiography (TTE) provides comprehensive information on cardiac chamber size (particularly left atrial size), wall thickness, and geometry, as well as quantitative measurements of ventricular systolic and diastolic function. It can also identify regional wall motion abnormalities that are suggestive of myocardial ischemia, and assess global ventricular performance using parameters such as ejection fraction and strain analysis. In addition, TTE enables the evaluation of cardiac valves in terms of morphology, motion, and hemodynamic function, allowing for the determination of stenosis or regurgitation severity.

Recent studies have indicated that left atrial enlargement may predispose individuals to AF [14,15]. Seko et al. demonstrated that left atrial enlargement (>40 mm) and reduced left ventricular ejection fraction (LVEF < 50%) were the strongest parameters associated with AF, surpassing left ventricular geometry [14]. In a previous study involving patients with isolated mitral valve disease, a significant correlation was identified between LA size and the occurrence of AF. AF was rare when the left atrial dimension was below 44 mm (3%), but prevalent when exceeding 40 mm (54%), highlighting the potential clinical utility of left atrial size in decisions regarding prophylactic anticoagulation and elective cardioversion [16].

Current Doppler echocardiography guidelines recommend assessing diastolic function by considering the ratio of early-to-late diastolic transmitral flow velocities (E/A) and by using the ratio of early diastolic mitral inflow velocity to early diastolic mitral annular tissue velocity (E/e′) as an indicator for estimating left ventricular filling pressures [17]. In patients with ESUS who underwent insertable cardiac monitoring, a high E/e′ ratio (≥8.65) was independently associated with AF detection after adjustment for age and sex [18].

### 2.6. Single-Lead Wearable Patch

An innovative, adhesive single-lead ECG patch (mobiCARE-MC100™; Seers Technology, Seongnam-si, Gyeonggi-do, Republic of Korea) weighing only 9.2 g and requiring no supplementary components has been developed for the identification of arrhythmias. This device enables uninterrupted, continuous ECG monitoring and offers greater comfort compared to conventional Holter monitors. The 24 h Holter monitoring device is relatively bulky, less comfortable for patients, and may interfere with daily activities, leading to reduced compliance.

Users can review their ECG recordings via a mobile phone application, and data are transmitted seamlessly to a central laboratory. Utilizing an artificial intelligence–driven algorithm, this single-lead ECG patch robustly classifies and analyzes data, thereby enhancing diagnostic accuracy. Notably, the device’s superior signal fidelity—stemming from its effective mitigation of motion artifacts—significantly aids in the precise detection of heartbeats [19]. ECG data are transmitted through a secure server managed by the device manufacturer. Clinicians interpreting both Holter and patch ECGs are blinded to clinical information.

Cardiac rhythm and heart rate variability data were obtained using the single-lead wearable patch (mobiCARE-MC100™). Continuous electrocardiographic monitoring with this device allowed for the detection of arrhythmias, including atrial fibrillation, premature atrial and ventricular contractions, and pauses. Average heart rate, heart rate trends, and episodes of tachycardia or bradycardia were also recorded and analyzed.

These data are accessible only to authorized personnel responsible for patient care or individuals granted approval by the Institutional Review Board (IRB) for the study’s intended use. Importantly, the device manufacturer did not participate in the trial design, data collection, or analysis.

### 2.7. Concomitant Application

At baseline, upon enrollment, AF detection rates are concurrently compared using a single-lead wearable patch and a 24 h Holter monitor, with observations spanning 48 h. The Holter monitor is used for 24 h (at least 18 h), whereas the single-lead wearable patch is applied for 5 days (at least 72 h). Patients with ESUS will undergo follow-up assessments using both the single-lead patch and the 24 h Holter monitor at enrollment (visit 0) and at 6 months (visit 1).

In the context of clinical AF, this study follows a specific definition: AF is identified when an ECG recording captures an episode lasting 30 s or longer. This definition aligns with the criteria established in the 2020 European Society of Cardiology guidelines for the diagnosis and management of AF [6].

### 2.8. Clinical Monitoring

During the 6-month follow-up period, both AF detection rates and the occurrence of recurrent stroke will be evaluated. Recurrent stroke will be confirmed by brain MRI or CT imaging.

### 2.9. Sample-Size Estimates

The sample-size calculation is based on the primary endpoint: “the detection probability for each group.” The detection probabilities were estimated as follows: standard treatment at 2.8% and automated continuous ECG monitoring at 15.8%, based on prior studies [20]. To establish the non-inferiority of the single-lead patch monitor with a significance level (alpha) of 5% and a power (1-beta) of 80%; each group requires 55 patients, considering a 20% drop-out rate. The calculation was performed using the Power and Sample Size website (http://powerandsamplesize.com/Calculators/Compare-2-Proportions/2-Sample-Non-Inferiority-or-Superiority, accessed on 22 November 2021). Given that this study adopts an intention-to-treat approach, our intended analysis includes cases of participant drop-out, excluding situations where a subject actively requests data removal. The first enrollment of the AVANT-GARDE commenced in April 2023, and the recruitment ended in 2024 with 116 participants.

### 2.10. Statistical Analysis

For baseline variables, bivariate relationships will be examined by employing McNemar’s test for categorical variables and paired samples *t*-tests or Wilcoxon signed-rank test for continuous variables, presented as either means with standard deviations or medians with interquartile ranges, depending on their distribution. The primary outcome will be analyzed comparatively between the single-lead monitoring group and the control arm using McNemar’s test for paired binary outcomes. Effect sizes will be presented as risk differences with corresponding 95% confidence intervals, derived from the discordant pairs. This approach directly reflects the open-label paired design of the trial.

During the 12-month observation period, Kaplan–Meier curves will be generated for each arm and compared using a log-rank test to evaluate potential differences. The Statistical Package for the Social Sciences (SPSS version 26.0, IBM SPSS Statistics, Armonk, New York, NY, USA) will be employed for statistical analyses. A *p*-value < 0.05 is deemed statistically significant.

## 3. Study Outcomes

**Figure 1:**
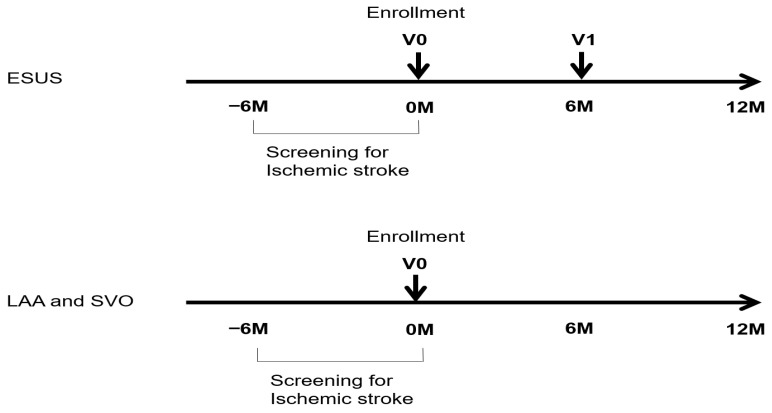
Study flow diagram. Abbreviation: ESUS = embolic stroke unknown source; LAA = large artery atherosclerosis; SVO = small vessel occlusion.

The primary outcome was the detection of AF using a single-lead wearable patch compared to a 24 h Holter monitor during the 6-month follow-up period (baseline visit: V0; 6-month visit: V1) in patients with ESUS. The secondary outcome was the comparison of AF detection rates between the two monitoring devices in stroke patients with large artery atherosclerosis (LAA) or small-vessel occlusion (SVO). The tertiary outcome was the occurrence of recurrent stroke among patients with ESUS, given that AF is infrequently diagnosed in individuals who experience multiple recurrent ESUS events (Figure 1).

## 4. Discussion

Unlike prior studies that primarily focused on prolonged monitoring in general stroke populations, this trial uniquely investigates early AF detection not only in patients with ESUS, but also in those with LAA and SVO. Even when physicians classify a stroke as LAA or SVO based on brain imaging, paroxysmal AF (pAF) may remain undetected. This study addresses a critical gap in optimizing AF detection strategies and has the potential to redefine approaches to secondary stroke prevention.

The AVERROES (Apixaban vs. Aspirin to Reduce the Risk of Stroke) study clearly demonstrated that anticoagulation therapy is generally more effective than antiplatelet therapy in preventing stroke and vascular embolic events in patients with AF. Consequently, detecting occult AF in cases of cryptogenic stroke holds significant clinical importance.

Previous studies have shown that prolonged ECG monitoring yields higher AF detection rates [21,22]. Implantable loop recorders (ILRs) have proven to be more effective in detecting AF than external loop recorders, with detection rates reaching as high as 41.4% after three years of ILR use [23]. However, the clinical application of ILRs remains limited due to their invasive nature and the indefinite duration of management associated with their use [24]. Current European Stroke Organization (ESO) guidelines recommend continuous cardiac rhythm monitoring for more than 48 h for AF screening in all patients with IS or TIA associated with ESUS and, if feasible, the use of ILRs to increase detection of occult AF [4]. Prolonged cardiac rhythm monitoring enhances the detection of subclinical AF, although the optimal duration remains uncertain. Therefore, when considering patient compliance, a device that is wearable for an extended period and allows for easy data collection would be particularly advantageous.

The single-lead ECG patch (mobiCARE-MC100™), characterized by its lightweight design and smartphone compatibility, enables continuous data acquisition and seamless transmission to a central system. According to the 2020 European Society of Cardiology (ESC) guidelines, such wearable single-lead ECG devices may be valuable for confirming a diagnosis of AF [6]. A recent systematic review and meta-analysis also recommended considering non-invasive rhythm monitoring as an initial approach before implementing more invasive methods [7].

Two large international randomized clinical trials—NAVIGATE ESUS and RESPECT ESUS—evaluated the efficacy of NOACs (rivaroxaban and dabigatran, respectively) compared to aspirin in patients with ESUS. However, both trials failed to demonstrate a benefit of NOACs over aspirin in preventing recurrent ischemic stroke [8,9]. Furthermore, the ATTICUS trial, which evaluated apixaban, was prematurely terminated following an interim analysis due to lack of efficacy [25]. Initially, it was hypothesized that covert AF frequently underlies ESUS and that NOACs might therefore be beneficial for secondary prevention. However, the lack of efficacy observed in the NAVIGATE ESUS and RESPECT ESUS trials suggests the need for more refined and selective inclusion criteria.

In this context, we sought to include patients exhibiting multiple indicators suggestive of covert AF, including embolic ischemic patterns on MRI as imaging markers, LA enlargement and E/e′ ratio as echocardiographic markers, and elevated NT-proBNP levels as serum markers. These multimodal biomarkers aim to enhance the detection of hidden AF.

In contrast to previous studies, the present trial uniquely explores early AF detection not only in patients with ESUS, but also in those with LAA and SVO. By addressing a key unmet need in identifying covert AF, this study has the potential to significantly influence strategies for secondary stroke prevention.

### Study Limitations

This study has several limitations. First, it is not a randomized controlled trial, but rather a non-blinded, prospective observational study; therefore, the possibility of detection bias cannot be excluded. Second, the accuracy and completeness of measurements largely depend on patient cooperation. Third, the relatively short follow-up period of six months may underestimate the rates of AF diagnosis and recurrent stroke incidence. Fourth, patients with ESUS will undergo follow-up evaluations at enrollment (visit 0) and at 6 months (visit 1). The 6-month interval may be too long, potentially resulting in missed episodes of paroxysmal AF (pAF). However, a recent meta-analysis of randomized controlled trials (RCTs) conducted between 2012 and 2023 investigating new AF detection after ischemic stroke (IS), and transient ischemic attack (TIA) identified two statistically significant time windows for new AF detection: 0–14 days and 6–12 months [26].

## 5. Conclusions

The European Society of Cardiology currently recommends the use of non-invasive patch-based ECG monitoring devices, which hold great promise for enhancing AF detection—a well-recognized cause of ESUS [6]. It is well established that prolonged cardiac monitoring increases the detection rate of paroxysmal AF. However, the optimal diagnostic method should be non-invasive, patient-friendly, and capable of maintaining high diagnostic accuracy. This new technology not only aims to reduce the burden on individual patients, but also has the potential to mitigate the broader socioeconomic impact by facilitating AF detection and preventing cardioembolic stroke, even in patients with non-cardiogenic stroke.

## Data Availability

Anonymized data will be shared upon reasonable request from any qualified investigator.

## Abbreviations

The following abbreviations are used in this manuscript:

AF	Atrial fibrillation
ESUS	embolic strokes of undetermined source
IS	Ischemic stroke
LAA	large artery atherosclerosis
SVO	Small vessel occlusion
pAF	Paroxysmal atrial fibrillation
LA	Left atrial
TEE	Transesophageal echocardiographic
MI	Myocardial infarction
MRI	Magnetic resonance imaging
ILR	Implantable loop recorders
